# Impact of the MooN Physical Education Program on the Socio-Emotional Competencies of Preadolescents

**DOI:** 10.3390/ijerph18157896

**Published:** 2021-07-26

**Authors:** Pablo Luna, Javier Cejudo, José A. Piqueras, Débora Rodrigo-Ruiz, Miriam Bajo, Juan-Carlos Pérez-González

**Affiliations:** 1 Department of Psychology, Faculty of Education, University of Castilla-La Mancha, 13071 Ciudad Real, Spain; pablo.luna@uclm.es; 2 Department of Health Psychology, Center for Applied Psychology, Faculty of Social and Health Sciences, Campus of Elche, Miguel Hernandez University of Elche (UMH), 03202 Elche, Spain; jpiqueras@umh.es; 3 Faculty of Education, International University of La Rioja (UNIR), 26006 Logroño, Spain; debora.rodrigo.ruiz@unir.net; 4 Department of Psychology, School of Medicine, University of Castilla-La Mancha, 13071 Ciudad Real, Spain; miriam.bajo@uclm.es; 5 Emotional Education Laboratory (EDUEMO Lab), National University of Distance Education (UNED), 28040 Madrid, Spain; jcperez@edu.uned.es

**Keywords:** child, adolescent, intervention, physical education, social and emotional learning, emotional education

## Abstract

Few studies have analyzed emotional educational experiences through physical education interventions. The objective of this study was to evaluate the effects on socio-emotional competencies of a physical education intervention (i.e., the MooN program) based on the instructional model known as the sports education model (SEM), compared to a physical education intervention based on the traditional model of direct instruction (TM-DI) in preadolescents. The sample consisted of 170 students between 10 and 13 years old (mean age: *M* = 10.76; standard deviation: *SD* = 0.73). Participants were randomly assigned to the experimental group (SEM; *n* = 87) and the active control group (TM-DI; *n* = 83). In the experimental group, the SEM-based intervention was applied, while in the active control group, an intervention based on the TM-DI was developed. A quasi-experimental design with repeated pre-test and post-test measures and an active control group was used. The self-efficacy inventory for multiple intelligences (IAMI-40) was used to assess the children’s socio-emotional competencies. The child perfectionism inventory was applied to evaluate the self-demand perfectionist efforts. The results confirmed that the MooN program (SEM intervention) promoted significant improvements in socio-emotional competencies. These findings support the potential of this physical education instructional model as an emotional education pathway for the socio-emotional improvement of preadolescent students.

## 1. Introduction

The WHO [1,2] recommends that schools function as an environment of prevention, intervention, and support for students’ mental health, promoting the development of the socio-emotional skills of school-age children as factors of covitality [3,4], and thus promoting social and emotional well-being [5,6]. Emotional education and health education converge on these goals, as does physical education.

A close empirical relationship has been observed among the improvement of emotional competence and training in respiratory patterns, in the regulation of heart rate variability, and in physical and sports exercise [7,8]. Thus, it seems likely that the development of physical and sports interventions in the educational context can help to optimize socio-emotional competencies that, in turn, contribute to prevent the development of maladaptive behaviors in young people and, in the same way, promote, both indirectly and directly, their personal and social well-being [9,10].

Hayat et al. [11] point out that the teaching and learning process could be a complex procedure in which socio-emotional factors are decisive. Likewise, interventions where social and emotional competencies play a key role at different educational levels are being promoted in the school setting [12]. Different studies show that these competencies are significant for improving the school environment [12,13,14] and are crucial to the quality of education [15].

Domitrovich et al. [13] assert that social and emotional learning (SEL) is: (a) a pedagogical process through which socio-emotional competencies are developed; (b) a fundamental multidimensional construct in the success of schools; and (c) an organizational framework that acts as a protective factor against the development of possible maladaptive behaviors in the students. Examining the different socio-emotional competencies, organized into the two domains of intrapersonal and interpersonal competencies, is a key factor in implementing effective educational interventions [13]. Similarly, authors such as Weissberg et al. [16] indicate that the child population could effectively acquire and apply their intrapersonal and interpersonal skills through SEL. Therefore, effective learning through SEL, in the classroom, involves the teaching and learning processes of socio-emotional competencies in intrapersonal and interpersonal interactions, favoring a meaningful school structure that promotes a positive and balanced comprehensive development of students [15]. In this sense, SEL is an essential component for success in life [16], which, when implemented in schools, fosters policies and practices that help students acquire and positively apply their knowledge, skills, and intrapersonal and interpersonal attitudes, as well as improve their personal and social well-being, protecting them against negative educational outcomes [14].

The so-called SEL programs are being applied more and more often in the school setting, especially in primary education (a critical period for academic participation and socio-emotional competencies) to promote social and affective skills in order to prevent negative behaviors and school failure [14,17]. Research indicates that active, participatory, and motivating interventions, when applied in school settings using the organizational approach of SEL, are significant and effective for promoting socio-emotional competencies, resulting in improvements in psychosocial and emotional adjustment [12,14,16,18]. Durlak et al. [19], in their meta-analysis, showed that educational interventions based on SEL were effective in improving the intrapersonal (emotional) and interpersonal (social) skills, behaviors and attitudes of students, in addition to creating an optimal social and emotional school environment with significant school achievement. Therefore, socially and emotionally competent students tend to be better integrated and adapted in the school and classroom, and can focus on academic tasks, enhancing their personal and social development [15,17,19].

Ren et al. [18] point out the importance of promoting intrapersonal and interpersonal factors in the school context through effective physical practices. In the same way, these authors assert that favoring intrapersonal motor skills (e.g., physical self-efficacy) and interpersonal skills (e.g., relationships and social support among students) with participatory and motivating experiences predicts positive integral development in students. The promotion of active habits with motor educational practices is a key requirement for the healthy development of children [20].

Bessa et al. [21] highlight that, within the school environment, physical education is recognized as a relevant academic discipline to promote students’ personal and social development, encouraging cooperative methodologies and positive socio-emotional experiences for students. Likewise, these physical and sports activities within physical education are beneficial for students from the integral perspective of the child [22,23]. There is some empirical evidence that physical education could be a significant psychoeducational intervention for developing effective forms of physical/sporting experiences, which will improve students’ socio-emotional competencies [24,25]. Physical education promotes a significantly flexible and cooperative environment favoring intrapersonal and interpersonal interactions among students [26]. Eime et al. [27] point out the positive synergy between the implementation of physical and sports practices with intrapersonal and interpersonal variables in school settings. Likewise, these authors establish that team and cooperative sports playing, in educational contexts, is associated with better socio-emotional health, due to the social nature of participation, and show improvements in children’s psychosocial well-being.

Quality physical education [28] should generate a school climate that favors the positive development of students in their different motor and socio-emotional skills. The current study is aligned with educational physical and sports practices that promote balanced growth in students and seek to improve their development in its different physical, social, and affective dimensions [29]. In the same way, evaluating the variables under study (e.g., interpersonal competence, intrapersonal competence, and self-demand perfectionist efforts) in the context of physical education is decisive for children’s socio-emotional improvement.

In this sense, perceived self-efficacy or degree of confidence, understood as the subjective perception of successfully carrying out activities related to intrapersonal and interpersonal factors, is also a relevant factor for students’ adequate social and emotional development [13,30]. On the other hand, regarding child perfectionism oriented toward him- or herself [31], it is a positive construct of the perfectionist effort, referring to the self-demand or perfectionist attitude with which the child faces his or her tasks [32,33]. Likewise, Stoeber [33] indicates that perfectionist efforts, understood as a dynamic multidimensional personality trait, could promote children’s healthy and adaptive educational settings. Méndez-Giménez et al. [34] point out the self-demand subscale as a positive indicator of perfectionism and determinant in schools, favoring the quality of life and personal and social well-being of students in the context of physical education. In the same way, the variable self-demand perfectionist efforts (e.g., self-oriented or intrapersonal perfectionism) have been related to adequate coping strategies, positive affectivity, and psychological adjustment [32,33]. Furthermore, students who present high self-demand levels in physical education maintain more positive interpersonal relationships [34].

In this previous context, promoting a methodological change is necessary for quality physical education [28]. A constructive academic discipline promotes motivating interventions [35] within a socio-emotional learning framework that evolves with effective pedagogical models, such as the physical education policy known as the sports education model (hereafter called the SEM) [36]. The SEM is understood within a pedagogical framework with the main objective that students: (1) have real physical and sports experiences, simulating an authentic structure and organization of sports games, all adapted to the curricular context of sports physical education; (2) acquire competence, enthusiasm, and a physical-sports culture [36]. It is an active and cooperative learning model centered on the student as an active subject, in contrast to the repetitive experiences and sports memory developed in physical education based on the traditional model of direct instruction (hereafter called the TM-DI) [29,37].

In recent years, the SEM has generated significant interest in scientific literature, with the publication of different reviews [38,39,40,41,42] and meta-analyses [43]. It is an effective teaching model that shows significant improvements in the educational context, in both students’ well-being [44] and socio-emotional development [25,37,40,45]. Similarly, it is an empirically validated model where recent findings, in line with the variables under study, show improvements in social indicators [25,37,45,46,47], as well as affective or emotional indicators [24,44,48].

Bessa et al. [38] note in a recent systematic review of the SEM that no research in primary education has been found that explicitly compares SEM-based interventions with TM-DI-based interventions. Other authors also highlight the importance of investigating the impact of SEM-based interventions in children [49,50]. In this sense, the present study aims to respond to this need to investigate the impact of SEM on early ages.

Taking into account the above, the purpose of this study was to evaluate, in preadolescents, the effectiveness of the MooN Program, which consists of a physical education intervention based on the SEM, in comparison with a physical education experience based on the TM-DI, with the variables: (1) interpersonal competence; (2) intrapersonal competence; and (3) self-demand perfectionist efforts. Three hypotheses were proposed, based on the fact that the application of the educational intervention (based on the SEM) will improve interpersonal competence (hypothesis 1), intrapersonal competence (hypothesis 2), and self-demand perfectionist efforts (hypothesis 3) in the participants of the experimental group.

## 2. Materials and Methods

### 2.1. Design

The study was carried out using a quasi-experimental design with repeated measures (pre-test and post-test), with an active control group. Participants were randomly assigned to the experimental group (EG) (MED-based intervention) and the control group (CG) (TM-DI-based intervention) using a cluster-randomized controlled trial.

The present study addressed the research problem by answering whether these programs (based on the SEM) effectively improve socio-emotional competencies in the school population, as stated by different authors in previous research [21,40]. This research aims to provide more empirical evidence of SEM-based interventions in the educational field, being congruent with recent critical and systematic reviews (e.g., [21]).

### 2.2. Participants

The total sample was obtained through a non-probabilistic incidental sampling method (convenience). It was made up of 170 preadolescents from an educational center located in an urban environment, aged between 10 and 13 years old (mean age (*M*) = 10.76; standard deviation (*SD*) = 0.73). Regarding the sociodemographic distribution of the sample: (a) by gender, 57% were girls, and 43% were boys; (b) by age, 40% were 10 years old, 45.9% 11 years old, 12.6% were 12 years old, and 1.5% were 13 years old.

**Figure 1:**
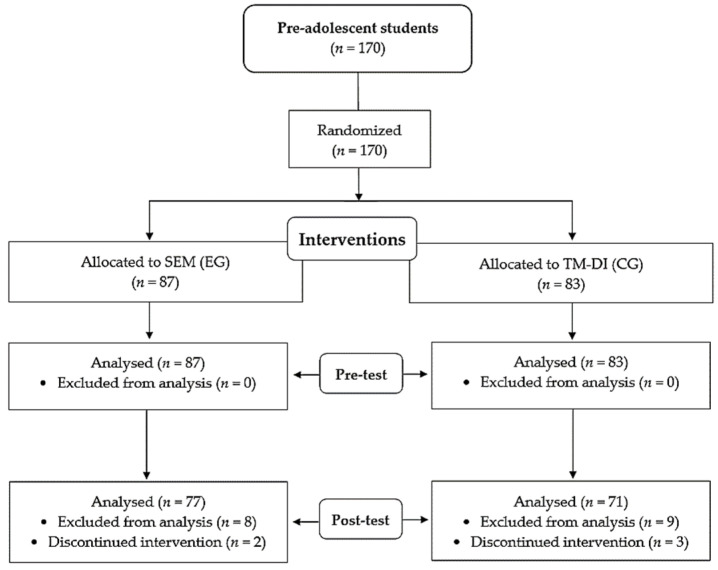
Flow diagram of interventions based on the sports education model (SEM; EG); physical education traditional model of direct instruction (TM-DI; CG).

The inclusion criteria (*n* = 148) in the study were: (1) written informed consent from the family (or legal guardian); (2) regular school attendance (≥ 85% face-to-face attendance); (3) active and regular participation in the educational intervention (≥ 85% of sessions). The exclusion criteria (*n* = 22) were: (1) students with more than 20% truancy; (2) failure to obtain the informed written consent from the family (or legal guardian); (3) not having taken any of the evaluation questionnaires (both before and after the intervention). Participant flow is displayed below (see Figure 1).

### 2.3. Instruments

In the present study, two measures were used to assess the variables under adequate psychometric parameters of reliability and validity.

#### 2.3.1. The Self-Efficacy Inventory for Multiple Intelligences (IAMI)

The surveying self-efficiency questionnaire for Spanish adolescents (IAMI-M40) [51], developed by Cejudo et al. [30], was used to evaluate the socio-emotional competencies. It consists of 40 items on a 10-point Likert scale (1 = “*I cannot perform this activity until—*”, to 10 = “*I am completely sure of being able to perform this activity successfully*”). However, in this study, only the following subscales of personal intelligence were used: (a) interpersonal self-efficacy (5 items), related to the ability to understand others’ personalities and to work effectively with them; and (b) intrapersonal self-efficacy (5 items), related to the ability to understand one’s own reasons, emotions, feelings and abilities. We refer to interpersonal competence as the ability to effectively manage and understand the concept of responsibility and social relationships in different contexts, promoting healthier and more positive interactions with others. In this way, this subscale encompasses the development of cognitive, social, and emotional skills in students and significant interpersonal behaviors (e.g., cooperative and team-playing behaviors and conversational skills). On the other hand, intrapersonal competence is defined as the emotional understanding of oneself and the ability to manage one’s thinking, enabling a precise representation and perception of oneself. Domitrovich et al. [13] point out that these subscales play a crucial role in students’ social and emotional learning, which can be understood as a process whereby students develop these competencies in intrapersonal (e.g., self-awareness, self-management) and interpersonal (e.g., social awareness, relationship abilities, and responsible decision-making) domains. The internal consistency, measured with Cronbach’s alpha and obtained through the specific sample of the current study, was α = 0.79 for intrapersonal self-efficacy and α = 0.75 for interpersonal self-efficacy.

#### 2.3.2. The Childhood Perfectionism Inventory (IPI)

The subscale of self-demand perfectionism from the IPI questionnaire (inventory of child perfectionism, validated in Spanish [32]) was used to assess perfectionist efforts oriented towards oneself in physical education. Self-demand items (8 items) report the perfectionist positive attitude with which the child faces his tasks (e.g., “*I do not like being the second, I want to be the first*”). This is a subscale understood as the development of positive impeccability efforts and effective performance standards. It is an indicator with a tendency to seek execution levels and significant results, assuming the positive criteria of efforts for themselves. Méndez-Giménez et al. [34] define the variable as a personality profile determining student motivation in the educational setting. Likewise, these authors point out that the self-demand factor is a healthy dimension of perfectionism. It consists of a 5-point Likert scale (1 = *strongly disagree*, to 5 = *strongly agree*). The internal consistency calculated through Cronbach’s alpha for self-demand was α = 0.70. These reliability results were obtained with the sample size of the current study.

### 2.4. Procedure

The research was developed in four stages. First, the MooN program was designed for the experimental group, to operationalize an SEM-based educational intervention. Second, the pre-test assessment was carried out in the experimental and control groups, administering the instruments with scheduled breaks to avoid student fatigue. Third, the educational SEM-based intervention on the experimental group and the TM-DI-based intervention on the control group were implemented simultaneously during physical education class. Fourth, after the interventions, the post-test assessment was carried out in both groups, with scheduled breaks to avoid student fatigue. A document with the date, name (coded numerically), gender, age, course, and a numerical code for each test to safeguard the participant’s anonymity and confidentiality, was delivered along with the instruments.

The study was developed, following the UCLM Ethics Code, under the international guidelines on experiments with human beings as described in the Nuremberg Code and the Declaration of Helsinki. As an investigation within an educational setting in primary education, it was authorized by the participating school center management team, the school council, and the teachers’ team. Similarly, the research team maintained constant telephone and telematic contact with the project’s management team, to supervise and coordinate the project. As an indispensable requirement for participating in the research project, written informed consent was delivered and requested to be signed by a family member (parent) or legal representative (legal guardian). Likewise, the ethical confidentiality requirements were respected per the voluntary and anonymous nature of the participants (ethical guidelines of the American Psychology Association (APA), [52], and Personal Data Protection Law from the Committee of Research Ethics on Human Beings, CEISH).

### 2.5. Educational Interventions

**Figure 2:**
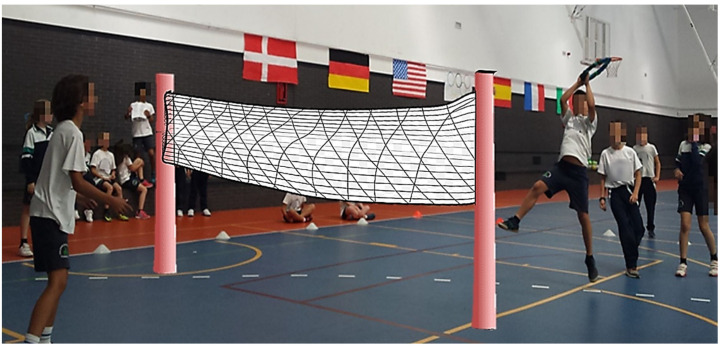
Real and modified team game with the selected sport (Polish ringo) on a sports court divided by a net (source: Luna, P.).

Two interventions were implemented in the physical education class with preadolescent primary education students: in the experimental group, the intervention followed the MooN Program, based on the sports education model (SEM) [36]; in the control group, the intervention was based on a didactic unit following a traditional model of direct instruction (TM-DI) [29]. Both were implemented simultaneously during school hours by specialist teachers in physical education. The same alternative and modified sports game (Polish ringo), understood as a divided-court or net sport, was used in both groups [53]. The implementation of a new and unknown sports game for the participants (i.e., Polish ringo) favored the inclusion and equality of opportunities in preadolescent students, with an equitable physical and sports practice in the development of the theoretical/practical sessions of physical education, as well as a better understanding of sports learning. All the students initially began with a similar understanding of the technical/tactical knowledge applied to this particular team sport. Likewise, alternative sports, such as Polish ringo, are efficiently adjusted to the school setting, being motivating, cooperative, flexible, socializing, and ultimately, positive for the students’ intrapersonal and interpersonal skills [53]. The sessions were held on a sports court divided into two by a central volleyball net, where the players had to receive, pass, and throw a mobile ringo hoop over the net, getting points when the hoop landed on the ground of the opposite team field (refer to Figure 2).

**Figure 3:**
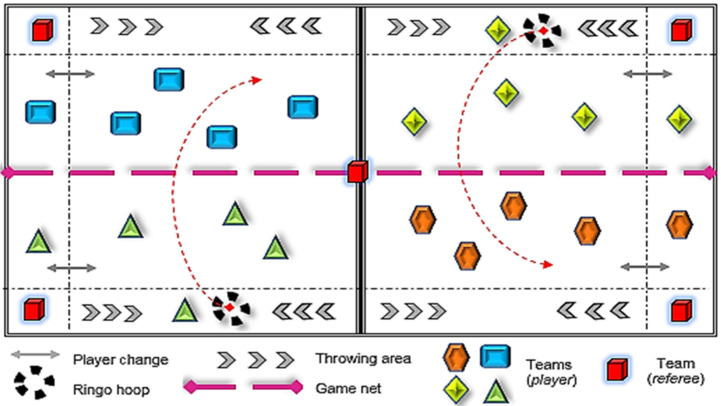
Ringo (Polish) sport practice session (source: Luna, P.).

The intervention was managed and controlled by external advisors, whose role and specific responsibility was to guide the research and the methodological sessions of the program. Likewise, there was no direct action on the part of the external researchers. In this regard, Sinelnikov’s suggestions [54] were followed: (1) virtual and personal contact was made to analyze and solve problems that arose during the research; (2) random and regular inspections were conducted of the educational center; (3) weekly analysis and verification were conducted of the investigation process. In the same way, before starting the educational experience, the research team held different meetings with the school management team and the physical education teachers responsible for the interventions (SEM and TM-DI) to establish the objectives and other curricular and didactic elements to be implemented in the sessions. The physical education department teachers agreed to select ringo (Polish) as the sport to be applied in the experimental and control groups (refer to Figure 2 and Figure 3).

#### 2.5.1. MooN Program

The educational intervention implemented for the experimental group, applying an adaptation of the intervention published by Luna et al. [25], was a new intervention called the MooN program. It was designed according to the recommendations proposed by Hastie and Casey [55], as well as with the content structure of the SEM, for the authentic and didactic recreation of an institutionalized sports context [36]: 

(1) Season or sports education didactic unit, with a longer duration (18 didactic sessions) compared to the traditional physical education units, following a sports organization of regular, formal, healthy, and meaningful competition (e.g., fair play, respect, and tolerance toward the opposing team). 

(2) Belonging to a team or affiliation, where the work teams were selected and grouped in a heterogeneous way (concerning gender and motor skills), according to a cooperative and collaborative learning methodology; these groups or work teams (5 members per team) were kept constant throughout the season to favor the development of shared goals and enhance the group’s identity. 

(3) Formal and regular competition: to experience a motivating and functional sports experience, an intervention was developed to simulate a real sports season, as a relevant feature for the practical experience and technical-tactical knowledge of physical and sports activity.

(4) Responsibility roles: the students were able to develop different roles (e.g., coach, journalist, referee, etc.) facilitating experiencing the sporting encounter from different dimensions or points of view. 

(5) Data recording: the process, performance, and sports behaviors were evaluated, using positive feedback. 

(6) Culminating event or celebration: to conclude, a celebratory and didactic classroom climate was promoted, through an awards ceremony for individual and collective achievements by handing out different educational awards, handmade by the students (e.g., trophies, certificates, medals, etc.).

The theoretical and practical intervention took place during school hours, over 18 sessions of 45 min (with a frequency of two sessions per week), as a didactic unit of sports education (called a season), by two specialist teachers in physical and sports education. The teaching staff responsible for carrying out this intervention had ten years of teaching experience and three years of applying the SEM. The season was structured in different phases:

(1) Initial or introductory phase (sessions 1–4). The process was characterized by the presentation of the SEM (with digital and audiovisual support) and the selected sport (Polish ringo); explanation and delivery of didactic material that will be used (e.g., personalized folders with educational themes, match minutes, contingency contracts, match reports, etc.) to the students; teaching of the self-construction of the materials applied in the intervention (e.g., mobile ringo hoops, trophies, medals, etc.) and the subsequent production of this material by the students, cooperatively (permanent workgroups/teams); selection and creation of mixed teams (cooperative groups of 5 girls and boys per team) organized permanently throughout the season, assigning each team an equipment color, shield, and motto as distinctive signs, as well as a representative team name that the students themselves could choose, based on an educational theme selected by the teachers (e.g., names of vertebrate animals); random assignment of responsibility roles. Throughout the season, shared roles were distributed to all participants, either an active and participatory player role, or a referee role (responsible for recording time, minutes, and match reports, in addition to compliance with the game’s regulatory normative (with emphasis on fair play and respect for the opponent)). This role played a key part in the process for the students, favoring intrapersonal skills, such as understanding fair play, and fostering interpersonal skills, such as their empathy with the referee team when they acted as players. On the other hand, participants were also assigned other roles of responsibility, such as: (a) manager, captain, and coach of the team, whose functions were coordination and mediation; (b) journalist, responsible for information and management of a virtual didactic blog, previously created by the teachers for the sports event; (c) physical trainer, a sports coach whose function is to design, direct, and manage the warm-ups before and after the practice sessions, in addition to being responsible for the sports equipment used; and (d) party committee, the one who organizes and manages the celebratory event.
(2) Intermediate phase (sessions 5–16). The preseason was developed with training sessions (friendly ringo matches promoting fair play and respect for the opponent) and regular competition, simulating a real sports tournament format and system (Round Robin: 5 × 5 team matches). Similarly, in this phase, active and participatory sessions were applied, such as: (a) initial warm-ups and final stretches (led by the physical trainer role); (b) team and autonomous practices in learning technical skills and tactical awareness relevant to ringo play (e.g., coordination, passing, receiving, serving, moving, etc.); (c) functional application of tasks with the development of responsibility roles (e.g., referee mediating for fair play or fostering tolerant sports habits, such as shaking hands before and after matches); and (d) completion of the sessions with an interactive assembly for communication and active listening between teachers and students, through the use of positive feedback and meaningful learning.
(3) Final phase (17–18). In this phase, the final competition was held (with the teams classified in the same previous league format), and the celebration of the final event was held, with the presentation of different educational awards (e.g., certificates and medals) produced by the students.

#### 2.5.2. Intervention Based on the Traditional Model of Direct Instruction (TM-DI)

The educational intervention applied in the control group was developed through a traditional teaching-learning methodology of direct instruction [29]. Authors such as Metzler [29] support a teaching model that encourages an active role for the instructor, where teachers fully direct instructional decisions on educational content, as well as didactic actions, class management, responsibility, and student participation.

The educational experience (theoretical and practical) was carried out during school hours, employing a traditional didactic unit conducted by two teachers trained in physical education, following the usual curricular programming of the participating school physical education department. The teachers responsible for carrying out said intervention had 15 years of teaching experience in physical education and previous practice in the TM-DI.

The didactic unit was implemented with the same sport as the previous intervention, including activities and physical and sporting contents, developed through a methodology specifically focused on the efficient reproduction and repetition of ringo’s basic technical and tactical skills. Furthermore, the participating students did not organize themselves into permanent teams or workgroups, nor did they use responsibility roles. The intervention applied in the control group was based on: 

(a) Out-of-context game activities and situations, practiced individually or in pairs (no real sporting activity was experienced). 

(b) Highly structured learning contents that allowed a meaningful observation by the teachers, critically examining the students’ motor behavior and technical/tactical skills. The teachers applied corrective feedback on incorrect answers, reinforcing only the correct answers [29]. 

(c) More active and total control by the teachers regarding the tasks’ presentation, introduction and explanation, organization, and methodological structure, as well as evaluation. 

(d) Rhythm and timing imposed by the teacher, with an external, unidirectional, and outstanding teaching position toward the group of students.

(e) Generalized teaching/learning process (not individualized), centered on a directive and instructive teaching function (based on immediate success criteria), and on the passive performance of students, determined by following the teacher’s instructions.

(f) The use of sports equipment not handmade by the students (mobile ringo hoops provided by the school). 

Therefore, traditional teaching models such as TM-DI develop constant and frequent feedback from teachers to students, identified by some authors as an essential resource to provide the efficient correction of motor behavior in students [29].

Table 1 shows the most relevant educational indicators of both interventions, applied to the experimental group (SEM) and the control group (TM-DI).

### 2.6. Data Collection and Analysis

Following collection, data were analyzed with the SPSS software, version 25.0 (IBM Corp. 1989, 2017, Armonk, NY, USA). First, the normality of the variables under study was calculated with the Kolmogorov–Smirnov test, all of which were adjusted to the assumption of normality (analyses performed with a 95% confidence interval). Second, the reliability evidence was calculated using Cronbach’s alpha coefficient (α). Third, to determine the educational intervention effects, the following psychometric analyzes were performed: (1) descriptive analysis (*M* = mean, *SD* = standard deviation) and variance analysis (ANOVA) with each of the scores obtained through the instruments used during the pre-test phase; (2) descriptive analysis and analysis of covariance (ANCOVA, using the pre-test scores) with the post-test scores, (3) finally, the effect size of the differences was analyzed according to the statistical criteria established by Cohen [56] (*small* < 0.50; *medium* 0.50–0.79; *large* ≥ 0.80).

## 3. Results

### 3.1. Pre-Test Analysis

The results of the analysis of variance (ANOVA) in the pre-test phase confirmed that, before starting the educational intervention, there were no significant differences in the variables studied, except for the variable interpersonal competence, in which the control group presents scores that are significantly higher, with a small effect size (*d* = 0.35) (see Table 2).

### 3.2. Post-Test Analysis

**Figure 4:**
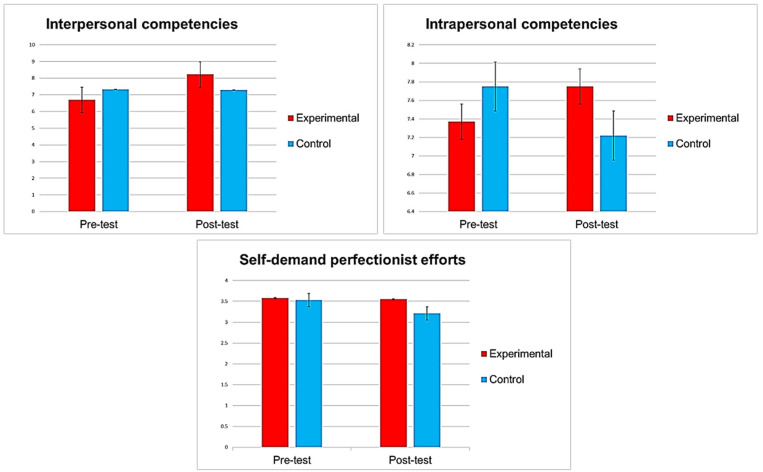
Significant effects of the MooN Program (SEM-based) in the experimental group.

The results analyzed in the present study in the post-test phase are collected in Table 2 and Figure 4.

#### 3.2.1. Evidence of Efficacy in Interpersonal and Intrapersonal Competencies

The post-test phase (ANCOVA) results showed significant improvement in interpersonal competence, with a medium effect size (*d* = 0.63) in favor of the experimental group. In favor of the experimental group, the results also confirmed significant improvement in intrapersonal competence with a small effect size (*d* = 0.44).

#### 3.2.2. Evidence of Efficacy in Self-Demand Perfectionist Efforts

After performing the covariance analysis (ANCOVA) in the post-test phase, the results showed a significant difference between the experimental and control groups in the variable self-demand perfectionist efforts, with a small effect size (*d* = 0.39). 

## 4. Discussion

The present study aimed to evaluate the effects of a physical education SEM-based intervention (i.e., MooN Program) in preadolescents, compared to a physical education TM-DI-based intervention, with the variables of interpersonal competence, intrapersonal competence, and self-demand perfectionist efforts.

In the first place, it should be noted that the results showed evidence of the efficacy of the intervention (SEM, experimental group) with significant improvements in interpersonal competence and intrapersonal competence. Therefore, Hypotheses 1 and 2 are confirmed.

The educational system should emphasize students’ personal and social development as a fundamental goal of current education [21,38]. Furthermore, in recent years, the scientific literature has been increasingly interested in teaching models that significantly impact students through high-quality learning environments [28]. Along these lines, with relevant empirical support, research promotes the SEM as an effective and positive model [38,39,40,41,43]. Similarly, physical and sports interventions within active educational environments (such as in physical education) that are effectively applied (SEM) are beneficial for students from a comprehensive perspective [22,23,29].

Our findings are consistent with other previous studies, which have shown that active, motivating, and healthy experiences, instrumentalized through effective and participatory teaching models such as SEM, are beneficial for students, both in their interpersonal [25,37,45,46,47] and intrapersonal dimensions [24,42,43,48]. Likewise, these results align with other authors, who have shown the efficacy of educational SEM-based interventions applied in physical education, with significant improvements in variables such as self-efficacy [37].

A possible explanation for these results could be the constructive and significant methodologies used in the intervention (e.g., cooperative learning, positive interdependence, decision-making, responsibility roles) contextualized from the SEM [36] and the SEL [13,15]. In this sense, the educational intervention implementation in preadolescents (a critical and sensitive, vital stage for the personal, social, and emotional development of students) with active and participatory experiences such as the one applied in the current study could promote socio-emotional competencies (interpersonal and intrapersonal) [18], and some motivational and autonomy indicators at an early age [38,42,43]. All this is favored by students’ participation in an alternative sport and team game, which, in line with other previous studies, improves their interpersonal skills [25,45] and relationship with classmates [46,47]. Eime et al. [27] point out that participatory sports games, developed as a team, are related to better psychosocial health. Similarly, sport applied cooperatively, as a form of educational, physical activity, improves the levels of psychological and social health in children.

Second, the results obtained did not confirm significant improvements in self-demand perfectionist efforts. Therefore, Hypothesis 3 is not confirmed. However, although there were no significant differences between experimental and control groups in the pre-test phase, the results in the post-test phase suggest that the MooN Program (SEM) could be acting as a protective factor before the significant decrease (between experimental and control groups) of the variable self-demand perfectionist efforts. In this sense, we need to point out that this positive indicator of child perfectionism [34], oriented to the search for beneficial standards of high performance and critical evaluation [31,32], represents a healthy factor in the educational setting to promote student well-being [34] and protection against negative [32] and maladaptive behaviors [33] in childhood. Furthermore, it is relevant to note that, at this stage, the prevention of socio-emotional problems is crucial [14,28]. In the same way, these results are convergent with those obtained in some systematic reviews, which conclude that the development of educational SEM-based interventions has shown improvements in variables such as student performance and commitment in the sports game [38], and in physical and sports technical-tactical competencies [41]. Likewise, these findings on self-demand could be supported by the idea that sports competition, together with cooperative learning generated by SEM in students, are crucial to favor their stimulation and motivation positively [38,42,43]. In this way, the present educational SEM-based intervention aims to promote a team sport that maximizes the responsibility of each participant in the achievement of shared objectives [57]. Siedentop et al. [36] consider that these physical/sports experiences are a beneficial tool to promote meaningful and autonomous learning by assigning and distributing individual and collective responsibility roles (e.g., referee, journalist, party committee, etc.).

These results regarding the self-demanding perfectionist efforts are probably related to the fact that preadolescents are more attracted to learning and improving their performance when they are actively motivated and are allowed to take part in decisions about learning a sports game experienced authentically, as well as being able to solve problems autonomously, resulting in a greater commitment to learning and a greater awareness of their difficulties and needs [38]. In the same way, the intervention based on the SEM could promote different socio-emotional competencies (e.g., self-management, social skills and responsible decision-making) relevant for success in the school setting [13,16]. In this sense, Méndez-Giménez et al. [34] point out that the self-demand variable is a positive personality trait of perfectionism, a determining factor in students’ well-being and motivation for learning, and a protective factor against demotivation in the educational context. Another possible explanation for these findings could be the structure and organization of competitive and physical/sports training in the intervention (SEM), implemented by seasons (long-term didactic unit). Authors such as Stoeber [33] point out that perfectionist efforts are positively related to task self-orientation and healthy performance execution, both in training and competition.

Despite these results, we must point out that this research has the following limitations: (1) the sampling is incidental, which may condition the generalization of the findings to other populations; (2) the exclusive use of self-reported instruments can increase the social desirability biases in preadolescents; (3) it would be convenient to carry out longitudinal studies to evaluate the impact of these interventions in the medium and long term [38]; (4) it would have been more useful to carry out a continuous evaluation during the development of the sessions; (5) the present study does not include qualitative methodology in the analysis of its impact [38]. Future research should increase the sample size, specifically analyze the effects of these psychoeducational interventions on students with special educational needs, such as students with special needs associated with physical disabilities, and evaluate the effects of these interventions in those preadolescents living in situations of sociocultural disadvantage or social exclusion.

## 5. Conclusions

A quality physical education [28] based on the SEM methodological principles, such as experiencing authentic sports and pedagogical experiences, affiliation or belonging, cooperative learning, and decision-making through responsibility roles [36], could significantly reinforce intrapersonal and interpersonal competencies in children [38], as well as their affective dimension [40]. The effective implementation of psychoeducational interventions, solidly based and with adequate methodological approaches, such as the SEM, could also positively favor students’ involvement during the teaching/learning process [38]. It is likely that, in line with the approaches outlined by Bessa et al. [38], these findings are a consequence of how the teaching model is: (a) defined (e.g., students as an active subject); (b) organized (e.g., the authentic recreation of the sports context); and (c) methodologically programmed (e.g., cooperative learning and the use of individual and group responsibility roles to enhance problem-solving and decision-making).

These results of improvements in socio-emotional competencies offer relevant educational and psychological implications for the educational community, as an essential aspect for the comprehensive development of students, in order to enhance personal and social well-being. The current study enriches the existing scientific literature, demonstrating that these types of programs, based on the SEM (e.g., MooN Program), promote viable and adequate pedagogical intervention strategies and methodologies to be developed in the school setting as effective socio-emotional learning [23,38,40] and, therefore, to improve and motivate the educational experience [43]. In this sense, the current study offers relevant practical implications, such as: (a) for the SEM, empirical support that encourages the development of innovative and motivating pedagogical approaches; (b) for teachers, an effective and promising teaching resource that could promote the involvement of students in their teaching and learning process; (c) for the students, efficient and motivating didactic tools (e.g., roles of responsibility, self-construction of materials) that benefit their intrapersonal (e.g., self-management) and interpersonal (e.g., cooperative work) skills and that favor their development socio-emotional in a positive way; and (d) for the educational context, active and participatory socio-emotional practices are promoted that help students to understand and apply intrapersonal and interpersonal knowledge, skills and attitudes [13,14,19].

This work extends the field of psychoeducational interventions to promote mental health and well-being by opening a new avenue for designing effective emotional education programs in the form of SEM-based physical education.

## Figures and Tables

**Table 1:** Characteristics of educational interventions in experimental and control groups.

	MooN Program Based on the Sports Education Model (SEM)	Traditional Direct Instruction Model (TM-DI)
Goal	Comprehensive student development (physical, social, and affective). Authentic physical/sports experiences, simulating a real structure and organization of an institutionalized sport, so that students acquire competence, enthusiasm, and physical/sports culture, becoming expert players who understand and value sport, distinguishing between positive and negative practices. Improvement of technical/tactical learning through a contextualized team sport (real and cooperative sports practice).	Segmented student development. Physical/sports experiences with highly structured sessions, based on the repetition of technical skills and essential tactical elements, favoring an efficient use of classroom time and resources, high rates of motor responses, and corrective feedback. Improvement in motor domains through the practice of a sport in a decontextualized way (sports practice in non-real game conditions).
Teachers’ role	Non-teacher-centered: constructive and cooperative learning where the teacher gradually favors and encourages greater responsibility in the student. Role of facilitator, moderator and guide in the teaching process and decision-making.	Teacher-centered: direct instruction. The teacher acquires an active role, drawing an educational scenario of practical application centered on his role. Unidirectional and instructional role of the teacher toward students.
Students’ role	Student-centered: an active role, where the practical application is centered on students. The student is participatory and actively involved in the teaching/learning process.	Non-student-centered: passive-receptive role, where students reproduce movements, actions, and content in a repetitive and automated way.
Task/Activity	Global and continuous approach to the activity from the beginning of the didactic unit (season).	Analytical and structured approach at the beginning to be global at the end of the didactic unit.
Methodology	Cooperative and competence-based learning. Positive interdependence. Use of responsibility roles. Cognitive involvement in decision-making, favoring reflection on action.	Traditional and expository learning (direct instruction). Direct control. Task assignment. Reproduces the model presented by the teacher, limiting student involvement.
Evaluation	Focused on the teaching-learning process continuously and globally. Formative evaluation.	Focused on the product or result of the teaching-learning process. Summative evaluation.

**Table 2:** Mean, standard deviation, variance analysis (ANOVA), covariance analysis (ANCOVA), and effect size for differences in means (Cohen’s *d*) in experimental and control groups.

	Pre-Test							Post-Test						
Measures	EG		CG		ANOVA			EG		CG		ANCOVA		
	*M*	*SD*	*M*	*SD*	*F*	*p*	*d*	*M*	*SD*	*M*	*SD*	*F*	*p*	*d*
**Socio-Emotional competencies**														
Interpersonal	6.70	1.89	7.32	1.63	5.563	0.019	0.35	8.22	0.73	7.29	1.94	22.142	0.000	0.63
Intrapersonal	7.37	1.99	7.75	1.56	1.987	0.160	0.21	7.75	0.91	7.22	1.45	10.031	0.002	0.44
**Perfectionist Efforts**														
Self-Demand	3.58	0.71	3.53	0.74	0.186	0.667	0.07	3.55	1.10	3.21	0.53	6.038	0.015	0.39

*Note*: EG = experimental group; CG = control group; *M* = mean; *SD* = standard deviation.

## Data Availability

The data presented in this study are available on request from the corresponding author. The data are not publicly available due to participants privacy.

## References

[1] World Health Organization [WHO] Local Action: Creating Health Promoting Schools. https://apps.who.int/iris/handle/10665/66576.

[2] World Health Organization [WHO] Mental Health Action Plan 2013–2020. https://www.who.int/publications-detail-redirect/9789241506021.

[3] Pérez-González J.-C., Yáñez S., Ortega-Navas C., Piqueras J.A. (2020). Educación emocional en la educación para la salud: Cuestión de salud pública. Clin. Health.

[4] Piqueras J.A., Rodriguez-Jimenez T., Marzo J.C., Rivera-Riquelme M., Martinez-Gonzalez A.E., Falco R., Furlong M.J. (2019). Social Emotional Health Survey-Secondary (SEHS-S): A Universal Screening Measure of Social-Emotional Strengths for Spanish-Speaking Adolescents. Int. J. Environ. Res. Public Health.

[5] Cefai C., Bartolo P.A., Cavioni V., Downes P. Strengthening Social and Emotional Education as a Core Curricular Area across the EU: A Review of the International Evidence. NESET II Report. https://www.salzburgglobal.org/fileadmin/user_upload/Documents/2010-2019/2018/Session_603/Strengthening-Social-and-Emotional-Education.pdf.

[6] Rougeaux E., Hope S., Viner R.M., Deighton J., Law C., Pearce A. (2020). Is Mental Health Competence in Childhood Associated with Health Risk Behaviors in Adolescence? Findings from the UK Millennium Cohort Study. J. Adolesc. Health.

[7] Laborde S., Dosseville F., Allen M.S. (2016). Emotional Intelligence in Sport and Exercise: A Systematic Review. Scand. J. Med. Sci. Sports.

[8] Laborde S., Eyre J., Akpetou J., Engler A.-C., Hofmann F., Klanderman J., Klein Y., Martins V., Reinhardt M.L., Zajonz P., Ruiz M.C., Robazza C. (2020). Emotional Competences Training. Feelings in Sport. Theory, Research, and Practical Implications for Performance and Well-Being.

[9] Halliday A.J., Kern M.L., Turnbull D.A. (2019). Can Physical Activity Help Explain the Gender Gap in Adolescent Mental Health? A Cross-Sectional Exploration. Ment. Health Phys. Act..

[10] McMahon E.M., Corcoran P., O’Regan G., Keeley H., Cannon M., Carli V., Wasserman C., Hadlaczky G., Sarchiapone M., Apter A. (2017). Physical Activity in European Adolescents and Associations with Anxiety, Depression and Well-Being. Eur. Child Adolesc. Psychiatry.

[11] Hayat A.A., Salehi A., Kojuri J. (2018). Medical Student’s Academic Performance: The Role of Academic Emotions and Motivation. J. Adv. Med. Educ. Prof..

[12] Durlak J.A., Domitrovich C.E., Weissberg R.P., Gullotta T.P. (2015). Handbook of Social and Emotional Learning: Research and Practice.

[13] Domitrovich C.E., Durlak J.A., Staley K.C., Weissberg R.P. (2017). Social-Emotional Competence: An Essential Factor for Promoting Positive Adjustment and Reducing Risk in School Children. Child Dev..

[14] Taylor R.D., Oberle E., Durlak J.A., Weissberg R.P. (2017). Promoting Positive Youth Development Through School-Based Social and Emotional Learning Interventions: A Meta-Analysis of Follow-Up Effects. Child Dev..

[15] Oberle E., Domitrovich C.E., Meyers D.C., Weissberg R.P. (2016). Establishing Systemic Social and Emotional Learning Approaches in Schools: A Framework for Schoolwide Implementation. Camb. J. Educ..

[16] Weissberg R.P., Durlak J.A., Domitrovich C.E., Gullotta T.P., Durlak J.A., Domitrovich C.E., Weissberg R.P., Gullotta T.P. (2015). Social and Emotional Learning: Past, Present, and Future. HANDBOOK of Social and Emotional Learning: Research and Practice.

[17] Calhoun B., Williams J., Greenberg M., Domitrovich C., Russell M.A., Fishbein D.H. (2020). Social Emotional Learning Program Boosts Early Social and Behavioral Skills in Low-Income Urban Children. Front. Psychol..

[18] Ren Z., Hu L., Yu J.J., Yu Q., Chen S., Ma Y., Lin J., Yang L., Li X., Zou L. (2020). The Influence of Social Support on Physical Activity in Chinese Adolescents: The Mediating Role of Exercise Self-Efficacy. Children.

[19] Durlak J.A., Weissberg R.P., Dymnicki A.B., Taylor R.D., Schellinger K.B. (2011). The Impact of Enhancing Students’ Social and Emotional Learning: A Meta-Analysis of School-Based Universal Interventions: Social and Emotional Learning. Child Dev..

[20] Reimers A.K., Boxberger K., Schmidt S.C.E., Niessner C., Demetriou Y., Marzi I., Woll A. (2019). Social Support and Modelling in Relation to Physical Activity Participation and Outdoor Play in Preschool Children. Children.

[21] Bessa C., Hastie P., Rosado A., Mesquita I. (2021). Sport Education and Traditional Teaching: Influence on Students’ Empowerment and Self-Confidence in High School Physical Education Classes. Sustainability.

[22] Amado-Alonso D., León-del-Barco B., Mendo-Lázaro S., Sánchez-Miguel P.A., Iglesias Gallego D. (2019). Emotional Intelligence and the Practice of Organized Physical-Sport Activity in Children. Sustainability.

[23] Bessa C., Hastie P., Araújo R., Mesquita I. (2019). What Do We Know about the Development of Personal and Social Skills within the Sport Education Model: A Systematic Review. J. Sports Sci. Med..

[24] Luna P., Guerrero J., Cejudo J. (2019). Improving Adolescents’ Subjective Well-Being, Trait Emotional Intelligence and Social Anxiety through a Programme Based on the Sport Education Model. Int. J. Environ. Res. Public Health.

[25] Luna P., Guerrero J., Rodrigo-Ruiz D., Losada L., Cejudo J. (2020). Social Competence and Peer Social Acceptance: Evaluating Effects of an Educational Intervention in Adolescents. Front. Psychol..

[26] Sánchez-Oliva D., Viladrich C., Amado D., González-Ponce I., García-Calvo T. (2014). Prediction of positive behaviors in physical education: A self-determination theory perspective. Rev. Psicodidáct..

[27] Eime R.M., Young J.A., Harvey J.T., Charity M.J., Payne W.R. (2013). A Systematic Review of the Psychological and Social Benefits of Participation in Sport for Children and Adolescents: Informing Development of a Conceptual Model of Health through Sport. Int. J. Behav. Nutr. Phys. Act..

[28] United Nations Educational, Scientific and Cultural Organization [UNESCO] Quality Physical Education (QPE): Guidelines for Policy Makers. https://unesdoc.unesco.org/ark:/48223/pf0000231101.

[29] Metzler M.W. (2017). Instructional Models for Physical Education.

[30] Cejudo J., Losada L., Pérez-González J.C. (2017). Inteligencias Múltiples y Su Relación Con Inteligencias Cognitiva y Emocional En Adolescentes. Univ. Psychol..

[31] Flett G.L., Hewitt P.L. (2002). Perfectionism: Theory, Research, and Treatment.

[32] Lozano Fernández L.M., García Cueto E., Martín Vazquez M., González L. (2012). Development and Validation of the Childhood Perfectionism Inventory (I.P.I.). Psicothema.

[33] Stoeber J., Childs J.H., Levesque R.J.R. (2011). Perfectionism. Encyclopedia of Adolescence.

[34] Méndez-Giménez A., Cecchini-Estrada J.A., Fernández-Río J. (2015). Perfectionism, Affectivity and Life Satisfaction in Physical Education. RICYDE Int. J. Sport Sci..

[35] Shields D.L., Funk C.D., Bredemeier B.L. (2018). Relationships among Moral and Contesting Variables and Prosocial and Antisocial Behavior in Sport. J. Moral Educ..

[36] Siedentop D., Hastie P., van der Mars H. (2020). Complete Guide to Sport Education.

[37] Pan Y.H., Huang C.H., Lee I.S., Hsu W.T. (2019). Comparison of Learning Effects of Merging TPSR Respectively with Sport Education and Traditional Teaching Model in High School Physical Education Classes. Sustainability.

[38] Bessa C., Hastie P., Ramos A., Mesquita I. (2021). What Actually Differs between Traditional Teaching and Sport Education in Students’ Learning Outcomes? A Critical Systematic Review. J. Sports Sci. Med..

[39] Hastie P.A., Martínez de Ojeda D., Calderón Luquin A. (2011). A Review of Research on Sport Education: 2004 to the Present. Phys. Educ. Sport Pedagogy.

[40] Evangelio C., Sierra-Díaz M.J., González-Víllora S., Fernández-Rio F.J. (2018). The Sport Education model in elementary and secondary education: A systematic review. Movimento.

[41] González-Víllora S., Evangelio C., Sierra-Díaz J., Fernández-Río J. (2018). Hybridizing Pedagogical Models: A Systematic Review. Eur. Phys. Educ. Rev..

[42] Chu T.L., Zhang T. (2018). Motivational Processes in Sport Education Programs among High School Students: A Systematic Review. Eur. Phys. Educ. Rev..

[43] Sierra-Díaz M.J., González-Víllora S., Pastor-Vicedo J.C., López-Sánchez G.F. (2019). Can We Motivate Students to Practice Physical Activities and Sports through Models-Based Practice? A Systematic Review and Meta-Analysis of Psychosocial Factors Related to Physical Education. Front. Psychol..

[44] Luna P., Rodríguez-Donaire A., Rodrigo-Ruiz D., Cejudo J. (2020). Subjective Well-Being and Psychosocial Adjustment: Examining the Effects of an Intervention Based on the Sport Education Model on Children. Sustainability.

[45] Kao C.C. (2019). Development of Team Cohesion and Sustained Collaboration Skills with the Sport Education Model. Sustainability.

[46] Menéndez-Santurio J.I., Fernández-Río J. (2016). Violence, responsibility, friendship and basic psychological needs: Effects of a Sport Education and Teaching for Personal and Social Responsibility program. Rev. Psicodidáct..

[47] Rocamora I., González-Víllora S., Fernández-Río J., Arias-Palencia N.M. (2019). Physical Activity Levels, Game Performance and Friendship Goals Using Two Different Pedagogical Models: Sport Education and Direct Instruction. Phys. Educ. Sport Pedagog..

[48] Méndez-Giménez A., Martínez de Ojeda D., Valverde-Pérez J.J. (2017). Emotional intelligence and motivational mediators in a season of Sport Education Mime. Ágora Para Educ. Física El Deporte.

[49] Layne T., Hastie P. (2016). Analysis of Teaching Physical Education to Second-Grade Students Using Sport Education. Education 3-13.

[50] Layne T., Hastie P. (2015). A Task Analysis of a Sport Education Physical Education Season for Fourth Grade Students. Phys. Educ. Sport Pedagog..

[51] Pérez E., Beltramino C., Cupani M. (2003). Inventario de Autoeficacia para Inteligencias Múltiples (IAMI). Fundamentos teóricos y estudios psicométricos. Evaluar.

[52] American Psychological Association [APA] Ethical Principles of Psychologists and Code of Conduct. https://www.apa.org/ethics/code/index.

[53] Méndez-Giménez A., Fernández-Río J., García-López L.M., González-Víllora S., Gutiérrez D., Martínez J., Sánchez R. (2011). Modelos Actuales de Iniciación Deportiva: Unidades Didácticas Sobre Juegos y Deportes de Cancha Dividida.

[54] Sinelnikov O.A. (2009). Sport Education for Teachers: Professional Development When Introducing a Novel Curriculum Model. Eur. Phys. Educ. Rev..

[55] Hastie P.A., Casey A. (2014). Fidelity in Models-Based Practice Research in Sport Pedagogy: A Guide for Future Investigations. J. Teach. Phys. Educ..

[56] Cohen J. (1988). Statistical Power Analysis for the Behavioral Sciences.

[57] Kolovelonis A., Goudas M. (2018). The Relation of Physical Self-Perceptions of Competence, Goal Orientation, and Optimism with Students’ Performance Calibration in Physical Education. Learn. Individ. Differ..

